# Supplementary material for: A neurobiological association of revenge propensity during intergroup conflict
Source: eLife. 2020 Mar 3;9:e52014. doi: 10.7554/eLife.52014 (PMC7058385; doi:10.7554/eLife.52014)
Supplement: Supplementary file 1. — This file shows the means (SD) and statistics for comparisons between the Revenge and Control groups. [file elife-52014-supp1.docx]

**Table S1**. Demographic information and psychological traits of participants of Revenge and Control groups in the fMRI experiment (Mean (SD)).

|  |  | | | Revenge group | Control group | F | p | η^2^_p_ |
| --- | --- | --- | --- | --- | --- | --- | --- | --- |
| Age |  | | | 23.27(2.76) | 23.84(2.16) | 1.13 | 0.291 | 0.013 |
| Education |  | | | 16.86(1.89) | 16.28(2.19) | 1.79 | 0.185 | 0.021 |
| Self-Esteem |  | | | 31.14(4.14) | 31.58(4.28) | 0.24 | 0.623 | 0.003 |
| Subjective Socioeconomic Status | | | | 5.36(1.51) | 5.37(1.48) | <0.01 | 0.979 | <0.001 |
| Intro-Extroversion |  | | | 11.98(5.48) | 13.26(4.96) | 1.30 | 0.257 | 0.015 |
| Self-Construal |  | | |  |  |  |  |  |
| Interdependent | | | | 59.39(9.39) | 61.26(6.99) | 1.11 | 0.296 | 0.013 |
| Independent | | | | 56.18(9.46) | 54.58(8.20) | 0.71 | 0.402 | 0.008 |
| Individualism-Collectivism | | | |  |  |  |  |  |
| Individualism | | | | 62.93(8.75) | 62.47(6.69) | 0.08 | 0.781 | 0.001 |
| Collectivism | | | | 73.20(11.41) | 75.35(7.84) | 1.04 | 0.311 | 0.012 |
| IRI | |  | | 64.00(11.06) | 66.16(9.03) | 1.00 | 0.321 | 0.012 |
| Aggression | | |  | 77.66(16.80) | 74.67(14.08) | 0.81 | 0.372 | 0.009 |

IRI = Interpersonal Reactivity Index
