# Supplementary material for: A neurobiological association of revenge propensity during intergroup conflict
Source: eLife. 2020 Mar 3;9:e52014. doi: 10.7554/eLife.52014 (PMC7058385; doi:10.7554/eLife.52014)
Supplement: Supplementary file 2. — This file shows the means (and SD) of emotions and attitudes and statistics for comparisons between the Revenge and Control groups. [file elife-52014-supp2.docx]

**Table S2.** Results of group manipulation check (Mean (SD)).

|  |  | **Revenge group** | |  | **Control group** | |  | Group* |  | Manipulation* |  | Involvement* |  | Group×Manipulation* |  |
| --- | --- | --- | --- | --- | --- | --- | --- | --- | --- | --- | --- | --- | --- | --- | --- |
|  |  | **In** | **Out** |  | **In** | **Out** |  | F, p, η^2^_p_ |  | F, p, η^2^_p_ |  | F, p, η^2^_p_ |  | F, p, η^2^_p_ |  |
| **Empathy** | **Involved** | 6.68  (2.04) | 5.93  (2.34) |  | 6.53  (2.09) | 6.53  (2.24) |  | F = 8.21  **p = .006**  η^2^_p_ = .095 |  | F = 0.06  p = .856  η^2^_p_ = .001 |  | F = 0.37  p = .657  η^2^_p_ = .005 |  | F = 1.08  p = .700  η^2^_p_ = .014 |  |
|  | **Uninvolved** | 6.55  (1.88) | 6.13  (2.13) |  | 6.58  (1.81) | 6.03  (2.03) |  |  |  |  |  |  |  |  |  |
| **Unpleasant** | **Involved** | 4.55  (2.22) | 4.15  (2.14) |  | 4.43  (2.43) | 3.55  (1.84) |  | F = 5.37  **p = .026**  η^2^_p_ = .064 |  | F = 0.43  p = .856  η^2^_p_ = .005 |  | F = 3.88  p = .424  η^2^_p_ = .047 |  | F = 0.88  p = .700  η^2^_p_ = .011 |  |
|  | **Uninvolved** | 3.98  (2.43) | 3.83  (2.15) |  | 3.98  (2.14) | 3.55  (1.95) |  |  |  |  |  |  |  |  |  |
| **Anger** | **Involved** | 2.85  (2.05) | 2.10  (1.41) |  | 2.70  (2.15) | 2.05  (1.43) |  | F = 9.68  **p = .005**  η^2^_p_ = .110 |  | F = 0.14  p = .856  η^2^_p_ = .002 |  | F = 0.58  p = .657  η^2^_p_ = .007 |  | F < 0.01  p > .999  η^2^_p_ < .001 |  |
|  | **Uninvolved** | 2.60  (1.96) | 2.23  (1.62) |  | 2.50  (1.87) | 2.05  (1.50) |  |  |  |  |  |  |  |  |  |
| **Fear** | **Involved** | 2.88  (2.29) | 2.35  (1.70) |  | 2.68  (2.32) | 2.10  (1.79) |  | F = 3.95  **p = .050**  η^2^_p_ = .048 |  | F = 0.39  p = .856  η^2^_p_ = .005 |  | F < 0.01  p = .954  η^2^_p_ < .001 |  | F = 0.05  p > .999  η^2^_p_ = .001 |  |
|  | **Uninvolved** | 2.63  (2.13) | 2.65  (1.98) |  | 2.40  (1.75) | 2.35  (1.72) |  |  |  |  |  |  |  |  |  |
| **Schadenfreude** | **Involved** | 1.60  (1.06) | 2.25  (1.78) |  | 1.55  (1.06) | 2.40  (1.92) |  | F = 14.91  **p <.001**  η^2^_p_ = .160 |  | F = 0.06  p = .856  η^2^_p_ = .001 |  | F = 0.61  p = .657  η^2^_p_ = .008 |  | F < 0.01  p > 0.999  η^2^_p_ < .001 |  |
|  | **Uninvolved** | 1.68  (1.10) | 2.25  (1.69) |  | 1.60  (1.34) | 1.98  (1.58) |  |  |  |  |  |  |  |  |  |
| **Like** | **Involved** | 5.15  (1.61) | 4.70  (1.60) |  | 5.55  (1.88) | 4.60  (1.89) |  | F = 23.57  **p <.001**  η^2^_p_ = .232 |  | F = 0.06  p = .856  η^2^_p_ = .001 |  | F = 2.06  p = .573  η^2^_p_ = .026 |  | F = 3.68  p = .236  η^2^_p_ = .045 |  |
|  | **Uninvolved** | 5.10  (1.61) | 4.58  (1.34) |  | 5.48  (1.83) | 4.18  (1.82) |  |  |  |  |  |  |  |  |  |
| **Trust** | **Involved** | 5.50  (1.47) | 4.73  (1.50) |  | 5.70  (1.81) | 4.30  (2.00) |  | F = 28.62  **p <.001**  η^2^_p_ = .268 |  | F = 0.90  p = .856  η^2^_p_ =.011 |  | F = 0.32  p = .657  η^2^_p_ = .004 |  | F = 4.28  p = .236  η^2^_p_ = .052 |  |
|  | **Uninvolved** | 5.40  (1.63) | 4.93  (1.02) |  | 5.50  (1.87) | 4.08  (1.97) |  |  |  |  |  |  |  |  |  |
| **Punish** | **Involved** | 2.18  (1.47) | 3.28  (2.06) |  | 2.15  (1.58) | 3.15  (2.11) |  | F = 27.19  **p <.001**  η^2^_p_ = .259 |  | F = 0.03  p = .856  η^2^_p_ < 0.001 |  | F = 1.57  p = .573  η^2^_p_ = .020 |  | F = 0.12  p > .999  η^2^_p_ = .001 |  |
|  | **Uninvolved** | 2.35  (1.49) | 3.05  (1.77) |  | 2.38  (2.00) | 3.43  (2.10) |  |  |  |  |  |  |  |  |  |
| *We conducted a repeated-ANOVA for each measurement with Involvement (Involved vs. Uninvolved targets) and Group (Ingroup vs. Outgroup targets) as the within-subject variables and Manipulation (Revenge vs. Control group) as the between-subject variable. All p values are reported after multiple comparison FDR correction. | | | | | | | | | | | | | | | |
