# Supplementary material for: A neurobiological association of revenge propensity during intergroup conflict
Source: eLife. 2020 Mar 3;9:e52014. doi: 10.7554/eLife.52014 (PMC7058385; doi:10.7554/eLife.52014)
Supplement: Supplementary file 3. — This files shows the results of a factorial analysis that tested the discriminant validity of the eight items related to measures of emotions and attitudes. The analysis revealed two factors, which explained 62.60% of total variance. Factor one was the emotion factor (explaining 37.65% of variance), which included five items: empathy (0.653), unpleasant (0.906), anger (0.748), fear (0.837), and schadenfreude (–0.349). Factor 2 was the attitude factor (explaining 24.95% of variance), which included two items: likability (0.907) and trust (0.918). [file elife-52014-supp3.docx]

**Table S3**. Factorial models of emotion and attitude rating items.

| No. | Item | Component 1 | Component 2 |
| --- | --- | --- | --- |
| 1 | Likability |  | .907 |
| 2 | Trust |  | .918 |
| 3 | Empathy | .653 |  |
| 4 | Unpleasant | .906 |  |
| 5 | Anger | .748 |  |
| 6 | Fear | .837 |  |
| 7 | Schadenfreude | -.349 |  |
| Explained variance (%) |  | 37.65 | 24.95 |

Rotation method: Varimax with Keiser normalization.
