# Supplementary material for: A neurobiological association of revenge propensity during intergroup conflict
Source: eLife. 2020 Mar 3;9:e52014. doi: 10.7554/eLife.52014 (PMC7058385; doi:10.7554/eLife.52014)
Supplement: Supplementary file 4. — This file shows the MNI coordinates of activated brain regions, cluster sizes, and Z values. [file elife-52014-supp4.docx]

**Table S4.** Brain activations elicited by painful vs. neutral expressions across the Revenge and Control groups.

| Region | | Cluster  Size | MNI Coordinates | | | Peak  Z |
| --- | --- | --- | --- | --- | --- | --- |
|  |  |  | x | y | z |  |
| All targets |  |  |  |  |  |  |
| mPFC/anterior cingulate | | 1007 | 6 | 53 | 34 | 6.49 |
| left MTC | | 693 | -57 | -55 | 4 | 5.83 |
| left occipital cortex | |  | -42 | -88 | 4 | 5.01 |
| left TPJ | |  | -63 | -43 | 34 | 4.99 |
| right MTC | | 879 | 54 | -31 | -5 | 5.75 |
| right TP | |  | 54 | 2 | -26 | 4.87 |
| right occipital cortex | |  | 36 | -94 | 1 | 4.80 |
| left IFG/AI | | 298 | -39 | 29 | -17 | 5.65 |
| right IFG/AI | | 388 | 45 | 32 | -5 | 5.45 |
| Precuneus | | 116 | -9 | -49 | 34 | 4.23 |
| Ingroup targets | | |  |  |  |  |
| left IFG/AI | | 328 | -42 | 29 | -14 | 6.66 |
| right MTC | | 824 | 54 | -28 | -8 | 6.41 |
| right occipital cortex | |  | 36 | -73 | -14 | 4.01 |
| mPFC | | 682 | 6 | 53 | 34 | 6.13 |
| right IFG/AI | | 270 | 48 | 29 | -5 | 5.68 |
| left MTC | | 799 | -60 | -61 | 16 | 5.50 |
| left TPJ | |  | -60 | -52 | 34 | 5.08 |
| left occipital cortex | |  | -45 | -85 | 1 | 5.01 |
| MCC | | 130 | -3 | -10 | 37 | 4.96 |
| Outgroup targets | | |  |  |  |  |
| mPFC | | 499 | -6 | 56 | 25 | 4.59 |
| mPFC: media prefrontal cortex; MTC: middle temporal cortex ; TPJ: temporoparietal conjunction; TP: temporal pole; AI/IFG: anterior insula/inferior frontal gyrus; MCC: middle cingulate cortex. Brain activations in response to painful vs. neutral expressions were identified by combining a voxel-level threshold of p < .001 and a cluster-level threshold of p < .05, FWE corrected. | | | | | | |
