# Supplementary material for: A neurobiological association of revenge propensity during intergroup conflict
Source: eLife. 2020 Mar 3;9:e52014. doi: 10.7554/eLife.52014 (PMC7058385; doi:10.7554/eLife.52014)
Supplement: Supplementary file 5. — This file shows the MNI coordinates of activated brain regions, cluster sizes, and Z values. [file elife-52014-supp5.docx]

**Table S5.** Brain activations elicited by painful vs. neutral expressions in the Revenge group.

| Region | | Cluster  Size | MNI Coordinates | | | Peak  Z |
| --- | --- | --- | --- | --- | --- | --- |
|  |  |  | x | y | z |  |
| All targets |  |  |  |  |  |  |
| mPFC/anterior cingulate | | 625 | 9 | 59 | 37 | 5.36 |
| right TP | | 168 | 33 | 20 | -26 | 4.18 |
| right IFG/AI | |  | 45 | 23 | -14 | 4.18 |
| Ingroup targets | | |  |  |  |  |
| right IFG/AI | | 328 | 48 | 29 | -8 | 5.13 |
| right TP | |  | 33 | 20 | -26 | 4.68 |
| mPFC | | 354 | 9 | 59 | 37 | 5.03 |
| left IFG/AI | | 156 | -42 | 29 | -14 | 4.82 |
| left TP | |  | -36 | 8 | -23 | 4.05 |
| right MTC | | 233 | 60 | -40 | -2 | 4.58 |
| MCC | | 120 | -3 | -10 | 37 | 4.20 |
| left MTC | | 100 | -57 | -61 | 7 | 4.13 |
| Outgroup targets | | |  |  |  |  |
| mPFC | | 150 | -3 | 53 | 31 | 3.87 |
| mPFC: media prefrontal cortex; MTC: middle temporal cortex ; TP: temporal pole; AI/IFG: anterior insula/inferior frontal gyrus; MCC: middle cingulate cortex. Brain activations in response to painful vs. neutral expressions were identified by combining a voxel-level threshold of p < .001 and a cluster-level threshold of p < .05, FWE corrected. | | | | | | |
