# Supplementary material for: A neurobiological association of revenge propensity during intergroup conflict
Source: eLife. 2020 Mar 3;9:e52014. doi: 10.7554/eLife.52014 (PMC7058385; doi:10.7554/eLife.52014)
Supplement: Supplementary file 6. — This file shows the MNI coordinates of activated brain regions, cluster sizes, and Z values. [file elife-52014-supp6.docx]

**Table S6.** Brain activations elicited by painful vs. neutral expressions in the Control group.

| Region | | Cluster  Size | MNI Coordinates | | | Peak  Z |
| --- | --- | --- | --- | --- | --- | --- |
|  |  |  | x | y | z |  |
| All targets |  |  |  |  |  |  |
| mPFC/anterior cingulate | | 434 | -9 | 47 | 25 | 5.01 |
| right MTC | | 330 | 51 | -28 | -5 | 4.95 |
| left IFG/AI | | 183 | -45 | 26 | -2 | 4.88 |
| left MTC | | 343 | -63 | -55 | 19 | 4.72 |
| left TPJ | |  | -54 | -61 | 34 | 3.81 |
| Ingroup targets | | |  |  |  |  |
| left MTC | | 260 | -60 | -58 | 19 | 5.33 |
| left TPJ | |  | -60 | -52 | 34 | 3.63 |
| right MTC | | 328 | 54 | -28 | -5 | 4.98 |
| mPFC | | 337 | -6 | 47 | 28 | 4.94 |
| left IFG/AI | | 208 | -54 | 29 | 10 | 4.82 |
| Outgroup targets | | |  |  |  |  |
| None. | |  |  |  |  |  |
| mPFC: media prefrontal cortex; MTC: middle temporal cortex ; TPJ: temporoparietal conjunction; TP: temporal pole; AI/IFG: anterior insula/inferior frontal gyrus; MCC: middle cingulate cortex. Brain activations in response to painful vs. neutral expressions were identified by combining a voxel-level threshold of p < .001 and a cluster-level threshold of p < .05, FWE corrected. | | | | | | |
