# Supplementary material for: A neurobiological association of revenge propensity during intergroup conflict
Source: eLife. 2020 Mar 3;9:e52014. doi: 10.7554/eLife.52014 (PMC7058385; doi:10.7554/eLife.52014)
Supplement: Supplementary file 7. — This file shows the statistical details of the moderation analysis that examined how group identity (Revenge vs. Control group) moderated the relationship between endogenous OT (Time-1) and mPFC activity in response to Involved_Ingroup target's pain. [file elife-52014-supp7.docx]

**Table S7.** Moderations of the relationship between OT-level at Time 1 and mPFC activity to Involved_Ingroup target's pain by Group (Revenge vs. Control group).

**Results with covariates**

**Model Summary**

R R-sq MSE F df1 df2 p

0.6171 0.3808 1456.5846 2.7206 11.0000 65.0000 0.0060

**Model**

coeff se t p LLCI ULCI

constant 47.7387 8.0495 5.9306 0.0000 31.6627 63.8148

Group -36.0284 9.9366 -3.6258 0.0006 -55.8732 -16.1836

MPFC 25.3401 7.4588 3.3973 0.0012 10.4437 40.2365

interaction -37.2011 16.4891 -2.2561 0.0274 -70.1322 -4.2699

Empathy -2.2344 4.7672 -0.4687 0.6409 -11.7553 7.2865

Unpleasant 4.5157 3.2399 1.3938 0.1681 -1.9549 10.9863

Anger -1.4001 3.4172 -0.4097 0.6834 -8.2248 5.4246

Fear 0.8608 4.5570 0.1889 0.8508 -8.2403 9.9619

Happy 1.1874 3.5591 0.3336 0.7397 -5.9207 8.2955

Like 2.6962 3.9440 0.6836 0.4966 -5.1806 10.5731

Trust -7.7793 4.9883 -1.5595 0.1237 -17.7416 2.1831

Close 0.5483 3.8502 0.1424 0.8872 -7.1410 8.2377

**Interaction: MPFC X Group**

R-square increase due to interaction(s):

R2-chng F df1 df2 p

Interaction 0.0543 5.0900 1.0000 65.0000 0.0274

**___________________________________________________________________________________**

**Results without covariates**

**Model Summary**

R R-sq MSE F df1 df2 p

0.5559 0.3090 1447.3807 10.8805 3.0000 73.0000 < 0.0001

**Model**

coeff se t p LLCI ULCI

constant 42.8914 4.3520 9.8555 < 0.0001 34.2177 51.5650

Group -38.5718 8.7117 -4.4276 < 0.0001 -55.9343 -21.2093

MPFC 26.3665 7.3078 3.6080 0.0006 11.8020 40.9310

interaction -40.9945 14.8215 -2.7659 0.0072 -70.5338 -11.4552

**Interaction: MPFC X Group**

R-square increase due to interaction(s):

R2-chng F df1 df2 p

Interaction .0724 7.6501 1.0000 73.0000 0.0072
