# Supplementary material for: A neurobiological association of revenge propensity during intergroup conflict
Source: eLife. 2020 Mar 3;9:e52014. doi: 10.7554/eLife.52014 (PMC7058385; doi:10.7554/eLife.52014)
Supplement: Supplementary file 8. — This file shows the statistical details of the moderation analysis that examined whether the mPFC activity mediated the relationship between endogenous OT (Time-1) and punishment tendencies towards the Involved_Outgroup target. [file elife-52014-supp8.docx]

**Table S8.** The results of the analysis of the mPFC activity mediating the relationship between endogenous OT (Time 1) and punishment tendencies towards Involved_Outgroup target.

| **Variable** | ***Coeff*** | ***SE*** | ***t*** | ***p*** |
| --- | --- | --- | --- | --- |
| **Regression Model 1 (Total effect of OT on punishment)** | | | | |
| Independent: OT | .157 | .167 | .938 | .355 |
| Dependent: punishment |  |  |  |  |
|  |  |  |  |  |
| **Regression Model 2 (OT to mPFC)** | | | | |
| Independent: OT | .500** | .146 | 3.412 | .002 |
| Mediator: mPFC |  |  |  |  |
|  |  |  |  |  |
| **Direct effect of mPFC on punishment** | | | | |
| Mediator: mPFC | .378* | .185 | 2.047 | .048 |
| Dependent: punishment |  |  |  |  |
|  |  |  |  |  |
| **Remaining direct effect of OT on punishment** | | | | |
| Independent: OT | -.032 | .185 | -.174 | .863 |
| Dependent: punishment |  |  |  |  |
|  |  |  |  |  |
|  | ***Coeff*** | ***SE*** | ***LLCI95*** | ***ULCI95*** |
| **Indirect effect of OT on punishment via mPFC (bootstrap result)** | | | | |
| mPFC | .189 | .110 | .009 | .441 |

** p<0.01, * p<0.05

Notes. Confidence intervals for indirect effect are bias-corrected and accelerated; bootstrap resamples = 5000; N = 37 for all tests.
