# Supplementary material for: A neurobiological association of revenge propensity during intergroup conflict
Source: eLife. 2020 Mar 3;9:e52014. doi: 10.7554/eLife.52014 (PMC7058385; doi:10.7554/eLife.52014)
Supplement: Supplementary file 10. — This file shows the means (SD) and statistics for comparisons between the Revenge and Control groups. [file elife-52014-supp10.docx]

**Table S10**. Demographic information and psychological traits of participants of Revenge and Control groups in the behavioral experiment (Mean (SD)).

|  |  | | | Revenge group | Control group | F | p | η^2^_p_ |
| --- | --- | --- | --- | --- | --- | --- | --- | --- |
| Age (years) |  | | | 22.50(2.75) | 21.56(2.23) | 2.75 | .101 | .035 |
| Education (years) |  | | | 15.95(2.22) | 15.15(2.18) | 2.58 | .112 | .032 |
| Self-Esteem |  | | | 28.98(6.65) | 30.26(5.54) | 0.86 | .356 | .011 |
| Subjective Socioeconomic Status | | | | 5.18(1.43) | 5.23(1.48) | 0.03 | .865 | <.001 |
| Intro-Extroversion |  | | | 13.90(5.04) | 13.41(5.53) | 0.17 | .682 | .002 |
| Self-Construal |  | | |  |  |  |  |  |
| Interdependent | | | | 61.23(5.86) | 61.08(7.60) | 0.01 | .923 | <.001 |
| Independent | | | | 59.50(8.88) | 60.03(7.55) | 0.08 | .778 | .001 |
| Individualism-Collectivism | | | |  |  |  |  |  |
| Individualism | | | | 64.32(6.97) | 63.33(7.92) | 0.35 | .556 | .005 |
| Collectivism | | | | 73.40(6.03) | 75.77(8.55) | 2.03 | .158 | .026 |
| IRI | |  | | 66.30(9.16) | 65.44(10.01) | 0.16 | .690 | .002 |
| Aggression | | |  | 76.73(15.56) | 76.00(19.65) | .033 | .856 | <.001 |
