# Supplementary material for: A neurobiological association of revenge propensity during intergroup conflict
Source: eLife. 2020 Mar 3;9:e52014. doi: 10.7554/eLife.52014 (PMC7058385; doi:10.7554/eLife.52014)
Supplement: Supplementary file 11. — This file shows the means (SD) and statistics for comparisons between the Revenge and Control groups. [file elife-52014-supp11.docx]

**Table S11**. Ingroup favoritism in self-report of emotions, attitudes, punishment tendencies, and punishment decisions in the new behavioral experiment (Mean (SD)).

|  | Ingroup | Outgroup | F | p | η^2^_p_ |
| --- | --- | --- | --- | --- | --- |
| Likability | 8.23(1.45) | 7.41(1.74) | 22.13 | < 0.001 | 0.223 |
| Closeness | 4.87(0.88) | 3.14(1.28) | 148.28 | < 0.001 | 0.658 |
| Impression | 8.56(1.34) | 7.90(1.58) | 16.58 | < 0.001 | 0.177 |
| Punishment tendency | 3.18(2.19) | 1.95(1.80) | 34.58 | < 0.001 | 0.310 |
| Punishment decision | 3.59(2.18) | 2.35(1.91) | 31.52 | < 0.001 | 0.290 |
